# Long-Term Impacts of Forest Ditching on Non-Aquatic Biodiversity: Conservation Perspectives for a Novel Ecosystem

**DOI:** 10.1371/journal.pone.0063086

**Published:** 2013-04-30

**Authors:** Liina Remm, Piret Lõhmus, Mare Leis, Asko Lõhmus

**Affiliations:** 1 Department of Zoology, University of Tartu, Tartu, Estonia; 2 Department of Botany, University of Tartu, Tartu, Estonia; 3 Department of Botany, Estonian University of Life Sciences, Tartu, Estonia; Lakehead University, Canada

## Abstract

Artificial drainage (ditching) is widely used to increase timber yield in northern forests. When the drainage systems are maintained, their environmental impacts are likely to accumulate over time and along accompanying management, notably after logging when new forest develops on decayed peat. Our study provides the first comprehensive documentation of long-term ditching impacts on terrestrial and arboreal biodiversity by comparing natural alder swamps and second-generation drained forests that have evolved from such swamps in Estonia. We explored species composition of four potentially drainage-sensitive taxonomic groups (vascular plants, bryophytes, lichens, and snails), abundance of species of conservation concern, and their relationships with stand structure in two-ha plots representing four management types (ranging from old growth to clearcut). We found that drainage affected plot-scale species richness only weakly but it profoundly changed assemblage composition. Bryophytes and lichens were the taxonomic groups that were most sensitive both to drainage and timber-harvesting; in closed stands they responded to changed microhabitat structure, notably impoverished tree diversity and dead-wood supply. As a result, natural old-growth plots were the most species-rich and hosted several specific species of conservation concern. Because the most influential structural changes are slow, drainage impacts may be long hidden. The results also indicated that even very old drained stands do not provide quality habitats for old-growth species of drier forest types. However, drained forests hosted many threatened species that were less site type specific, including early-successional vascular plants and snails on clearcuts and retention cuts, and bryophytes and lichens of successional and old forests. We conclude that three types of specific science-based management tools are needed to mitigate ditching effects on forest biodiversity: (i) silvicultural techniques to maintain stand structural complexity; (ii) context-dependent spatial analysis and planning of drained landscapes; and (iii) lists of focal species to monitor and guide ditching practices.

## Introduction

An increasing proportion of the Earth is covered by anthropogenically transformed ecosystems, which contain new combinations of species and have the potential for changed functioning [1]. Drainage of wetlands can produce such novel ecosystems through fundamental changes both in terrestrial and aquatic systems, notably in nutrient and hydrological dynamics, in the structure, functioning, quantity, and configuration of aquatic ecosystems *in loco* and downstream, and in soil properties resulting in enhanced plant growth that, in turn, modifies terrestrial heterotrophic biota and biogeochemical cycles [2], [3], [4], [5]. Draining (primarily for agriculture; [6]) has already transformed vast natural areas and many kinds of wetlands, particularly depression and slope wetlands [7].

Forested wetlands are extensively drained in many parts of the world. In the tropics, drainage typically accompanies forest clearing for food crops, oil palm and industrial timber plantations (e.g. [8], [9]), while northern temperate and boreal forested and semi-open wetlands are frequently drained for increasing timber yields and better access to timber resources. In Fennoscandia, Russia and the Baltic States over 13.5 million hectares of wetlands have been drained for forestry [10]; in Canada similar approaches are being considered for the near future [11], [12].

The influence of forest drainage on tree growth, timber production and greenhouse gas fluxes have been studied extensively (e.g. [4], [10], [13], [14]), but biodiversity assessments are extremely scarce (but see [2]). This contrasts with conservation planning practices that routinely assume that drainage impacts are severe and widespread. For example, the number of red-listed species, which are considered to be (potentially) threatened due to drainage of forests and open mires, exceeds 300 in Sweden [15] and 150 in Estonia (data extracted from [16]).

Biodiversity responses to forest drainage are apparently complex and result from various interacting changes in abiotic and biotic conditions. Aquatic biota respond rapidly to: increased amounts of solid sediments downstream of the drainage system [17], [18], [19]; reduced abundance, size, and diversity of natural bodies of water in the drained area [20]; and the appearance of ditches as a novel habitat (e.g. [21], [20], [22],). In the case of non-aquatic biota, short-term and long-term impacts differ. For example, birds such as cavity-nesting passerines [23], [24] or forest grouse [25] may experience increased predation soon after the ditching of wet forests because of water-level reduction and habitat fragmentation. The long-term impacts, which are the focus of the current study, become evident when the upper peat layers have been largely decomposed and the wetland converts to another, relatively stable ecosystem type–*decayed-peat forest* [26]. During this conversion, biological activity in the topsoil increases and a thick litter layer is formed; the profound changes that take place in vegetation [26], [27] subsequently influence heterotrophic organisms [28], [29]. The conversion also affects disturbance regimes, for example, by increasing fire frequencies [30] and reducing floods in the forest.

One could assume that the non-aquatic organisms most vulnerable to draining are moisture-dependent species, notably those depending on atmospheric relative humidity and lacking mechanisms to prevent desiccation (wetland species of green algae, cyanobacteria, lichens and bryophytes) or those having high body content of water and a permeable integument (land snails). Additionally, those species that have special traits for tolerating flooding may be replaced by generalists, less flood-tolerant alien species, and native taxa typical of drier forests [2], [31], [32]. The new conditions may support some rare species absent from wetlands; for example, terrestrial orchids seem to benefit from the shade and nutrient release in northern decayed-peat forests [33]. Such species-specific responses collectively suggest that post-drainage species assemblages are unprecedented and qualify under the “novel ecosystem” concept (sensu [1]).

Decayed-peat forests merit applied biodiversity research for at least three reasons. First, following the decades of extensive draining (e.g. [34]), such forests now comprise large, and increasing areas (e.g., ≥10% of forestland in Finland; [35]). Because drained forests are usually dispersed across the landscape, the expanse of reserve networks inevitably includes them [36] and old ditches are commonly observed in long-protected stands that appear structurally “primeval” [37]. Secondly, decayed-peat forests are rather semi-natural than highly transformed ecosystems [38], with at least plant [27], [39] and bird species richness [40] comparable to their natural predecessor ecosystems. Thus, their role for biodiversity should be explored by distinguishing and monitoring particular threatened species that either remain from the original forest or are able to colonise drained sites from other ecosystems. Thirdly, draining is usually accompanied with other measures to increase timber yield, such as forest-road building [41] and clearcutting, with new pulses of draining activities following from the need to facilitate regeneration in waterlogged sites (e.g. [34]). Understanding both the drainage impacts *per se* and the complex impacts is essential for effective management prescriptions.

In the current study we provide the first comprehensive biodiversity assessment of long-term drainage impacts in northern swamp forests at scales relevant for management planning. The assessment is based on stand-scale surveys of four large terrestrial and arboreal taxonomic groups that are potentially sensitive to draining: vascular understory plants, bryophytes, lichenised and allied fungi, and snails. We analyse their assemblages to explore: (i) how they differ in natural and drained forests in terms of species richness and composition, and how those differences are related to stand-structural features; (ii) which species of conservation concern can inhabit decayed-peat forests and which ones disappear; and (iii) how species-scale and assemblage-scale conservation values of this novel ecosystem are expressed along the gradient of timber-harvest intensity. We provide the answers based on a set of standardised field surveys in differently managed stands (old growth; mature commercial forests; low-level retention-cut areas; and clearcuts) in natural swamp and decayed-peat areas in Estonia, northern Europe.

## Materials and Methods

All necessary permits were obtained for the described field studies. The National Environmental Board issued the permissions to work in reserves. No specific permits were required outside reserves (all the lands were state-owned).

### Study Area and Study Design

**Figure 1:**
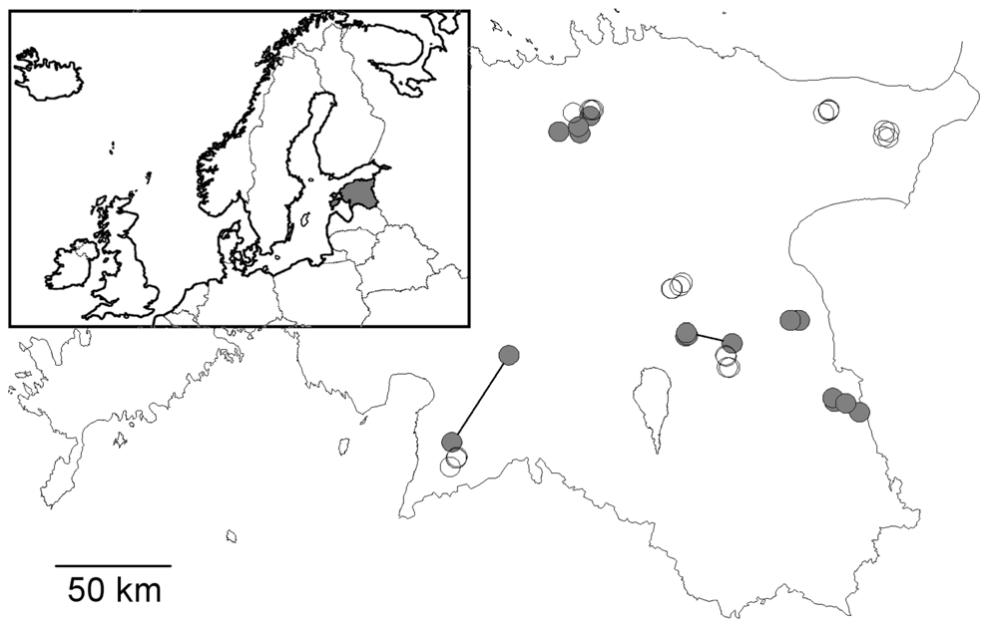
Locations of the study plots in Estonia. Filled circles–natural swamps, empty circles–drained sites. Each cluster of plots of similar type contains four differently managed plots (a ‘block’ of treatments) typically <10 km from each other; the connecting lines indicate two swamp ‘blocks’ where cutover plots were located farther away.

The study was carried out in 44 plots (22 forests and 22 cutover sites) in the Estonian mainland (Fig. 1). Estonia is situated in the European hemiboreal vegetation zone [42]. The mean air temperature is 17°C in July and −6.5°C in January and the average precipitation is 600–700 mm/yr. The terrain is flat and all the study plots were situated <100 m above sea level. Forest drainage (ditching) started in the 1820s in Estonia, and became large-scale and mechanised in the 1950s (10,000–20,000 ha drained annually; [43]). Currently, over 0.3 million ha of forest stands are classified as decayed peat type [44] and artificial drainage systems encompass over 0.6 million ha of the total 2.2 million ha of forestland. Drainage is practiced all over the forestland, although only reconstruction of existing drainage systems is allowed in the FSC-certified state forests (36% of all forests). In recent years, such reconstruction has been affecting about 20,000 ha annually (K. Kohv/State Forest Management Service, pers. comm.).

The study plots were arranged according to the principles of block design and they were selected as a part of a larger study representing several site types (see [45], [46] for details on plot selection). Each “block” included four 2-ha plots (four management types on one site type) in one forest region (a contiguous forest area on similar bedrock and terrain, and having a similar management history), and each plot represented a different stand. Because of the long-term management focus, the plot locations could not be assigned randomly or follow an experimental block design as their availability was determined by historical management. However, the plots were well dispersed (Fig. 1) and various principles were followed to assure their comparability. To reduce landscape effects, the plots within each block were established as close as possible to each other, and blocks of both site types were studied within the same region if present. Immediate edge effects were avoided by locating the plots in the central parts of the stands (at least 5–10 m from adjacent stands). Rectangular areas were preferred, but in some cases topography and stand configuration led to more complex shapes.

This study focuses on the comparison of two site types: mobile-water swamp forests in natural hydrological condition (hereafter: swamp sites; replicated as five blocks) vs. those converted to the decayed peat type (hereafter: drained sites; replicated as six blocks; Fig. 1). The swamp sites were located on thin flooded well-decomposed eutric Histosols and Fluvisols, with a peat layer ≥30 cm (pH_KCl_ 5.0–6.5), in lowlands and valleys along rivers or around bogs. These seasonally waterlogged forests constitute a distinct and threatened ecosystem [47], [48] and are characterised by abundant black alders (*Alnus glutinosa*) in the tree layer. The drained sites (*Oxalis* type) had well decomposed peat soils (pH_KCl_ 4.0–6.5)–residuals of the original swamps–but their current deciduous tree component had already been established on drained soil (i.e. the ditching impact had lasted for >50 years). Their comparability to the natural swamp sites was established based on topographic position, the incidence of floods in spring, tree species composition (notably lack of *Pinus sylvestris* characteristic of mixotrophic and ombrotrophic sites), and the wetland status on historical maps.

The four management types within each block represented timber-harvesting intensity: (i) old growth (the coniferous component 115–180 years old; stand ages up to at least 300 years; no visible signs of harvesting); (ii) mature (65–90 years old) semi-natural commercial forest (both recently thinned and unthinned); and naturally regenerating clearcuts (usually 6–11 years post-cut); (iii) with; and (iv) without retention trees. The retention cuts (management type iii) had on average 11 m^3^ ha^–1^ of live retention trees (range 2–29 m^3^ ha^–1^), which, however, produced on average only 4% canopy cover (maximum 11%).

### Data Collection

The field surveys of vascular plants, bryophytes and lichens followed the fixed-area, fixed-effort approach [49], as adjusted and standardised for Northern European conditions (e.g. [50]). Each of these three taxonomic groups was surveyed separately by the same observer all over the stand for four hours in a suitable season between 2005 and 2010 (snow-free season for lichens and bryophytes surveyed by P. L.; July–August for vascular plants surveyed by A.L.). In the most species-rich assemblages (e.g., lichens in herb-rich old forests), such an effort enables approximately half of all species present in a 2-ha stand to be discovered [51], while the species lists can be near-complete for conspicuous and less diverse groups [33]. Hence the assemblage differences detected should be considered conservative.

We used a five-point frequency scale of lichen and bryophyte species abundance based on the number of records (each record referring to a distinct substrate item): one record (1); 2–5 records (2); 6–15 records (3); 16–100 records (4); and >100 records (5). For herbaceous plants, we used a ten-point abundance scale, ranging from one shoot (score 1) or 2–3 scattered shoots or a clone (score 2) to local dominance (score 8) or total dominance (score 9 for <80% total cover, score 10 for >80% cover). In addition to lichenised fungi, “lichens” also included lichenicolous and some saprotrophic fungi (such as calicioids) traditionally surveyed by lichenologists. Cryptogam (lichen and bryophyte) specimens not identifiable in the field were collected for routine laboratory examination, using microscopes and the thin-layer chromatography method to detect lichen compounds.

For the snail survey, 3 litres of litter and topsoil, passed through a sieve with a 1 cm mesh, were collected from each plot once in August–September 2008 or in September 2009. The material was collected as six 0.5-litre subsamples, each obtained by haphazardly sampling different microhabitats while walking slowly in the forest; that volume method was combined with a simultaneous visual search (see [52]). The samples from one block of plots were collected in a short time period, usually on the same day; due to these technical constraints the north-easternmost drained block (Fig. 1) was not sampled. When identifying species in the lab, we also distinguished juveniles and adults, live individuals and empty shells. Seventy specimens of *Pisidium* bivalves found were included among snails as one taxon.

Taxonomy follows Kukk [53] for vascular plants, Santesson et al. [54] for lichens, Ingerpuu and Vellak [55] for bryophytes, and Kerney and Cameron [56] and Glöer and Meier-Brook [57] for snails. We distinguished *species of national conservation concern* (SPEC; listed in Tables S1, S2, S3, S4) as those: (1) on the Estonian Red List (categories RE, CR, EN, NT, VU and DD [16]); (2) rare or little known (up to 10 records in Estonia); or (3) established as old-growth indicators [58], [59], [60]. The criteria (2) and (3) were available or meaningful for lichens and bryophytes only. Reference materials are deposited in the mycology collections of the Natural History Museum of the University of Tartu (TUM; lichens) and in the herbarium of the Estonian University of Life Sciences (TAA; bryophytes).

The procedure of measuring stand structure has been described in detail by Lõhmus and Kraut [45]. In brief, we established, using a standard procedure, four straight 50-m transect lines in each plot. We then used a combination of: (i) area-based methods for estimating the densities of live and standing dead trees (≥10 cm diameter at breast height; including broken-top snags ≥1 m tall); (ii) the line-intersect method for volumes of downed logs (≥10 cm diameter at intersections with the line) by decay class, and the ground cover of bryophytes; and (iii) visual point estimates (at 10% accuracy) of canopy cover at 10-m intervals, along those lines. All standing dead trees are termed “snags” in this paper. We calculated Shannon indices of the species diversity of live trees (based on their numbers) and of decay-stage diversity of CWD (i.e., snags and logs; based on volume distribution among five decay stages). The latter was interpreted to indicate continuity of the CWD input in time. For the purposes of this study, only average estimates for each plot were used.

### Data Analysis

The main analyses addressed plot (2-ha) scale differences between swamp and drained sites, given that the latter originated from the former and assuming that such differences would mostly reflect long-term drainage impacts. In most analyses, hepatics and mosses were distinguished among bryophytes.

First, we established, using split-plot ANOVA, differences between swamps and drained plots (a factor variable) in the following stand-scale statistics: (i) total species richness and the number of SPEC in each taxonomic group; (ii) snail abundance (empty shells included); and (iii) stand structural features (Table S5). We treated a block as an independent observation with its four management types included as a within-subjects factor (the plots of a block can be viewed as parts of the same forest). We used ln and square-root transformations where appropriate to normalise distributions, and LSD post-hoc tests for detecting significant contrasts between groups. For two structural features, which did not meet normal distribution requirements even after the transformations, we used Mann-Whitney U-tests to compare swamps vs. drained plots separately within each management type (Table S5).

In a set of supplementary analyses, we explored which environmental factors (variables iii above) might explain the swamp vs. drained site differences in stand-scale species richness and snail abundance (i and ii above). These analyses included mature and old-growth forests only, because clearcutting created structurally distinct environments and that appeared to override any other effects (see Results). We first calculated correlation coefficients for each of several different environmental variables with each of the response variables (Table S6). The variables that appeared significant at p<0.05 were then pooled with site type in multifactor general linear models (Type III approach) to explore whether any of them could induce changes in the significance of the site type effect compared with that detected in ANOVA. We considered environmental factors of known biodiversity importance that: (1) are supposedly drainage-sensitive, such as canopy composition–densities of black alder and Norway spruce (*Picea abies*) and tree-species diversity [26], [61], [62]–and treefall abundance [63]; (2) maintain and/or indicate moisture (bryophyte cover [64]); (3) are highly logging-sensitive, such as the amount and continuity of dead wood and the abundance of late-seral tree species [45], [65]. Because snails can be influential herbivores [66], [67], [68], snail abundance was included as an environmental factor in lichen and plant analyses.

We tested the impacts of drainage on the assemblage composition of each taxonomic group using Multi-Response Permutation Procedures (MRPP). This procedure tests whether Sorensen (Bray-Curtis) distances among pre-defined classes exceed those resulting from random assignment of sample units to those classes, and it has the advantage of not requiring distributional assumptions that are seldom met with ecological assemblage data. Eight classes of sites were distinguished (two site-types×four management types) to compare drained to undrained for each management type separately, and among management treatments for each site type separately. For forests, we visualised the results using non-metric multidimensional scaling (NMS) based on the Sørensen index as the measure of dissimilarity in PC-ORD vers. 6.07 [69]. The medium autopilot mode was used to choose the number of dimensions and, after three-axis solutions were selected based on stress values, three sets of NMS with real data (250 runs each) were performed manually. The main data matrix (based on 40 plots) comprised, for each species, the number of specimens (in snails; water snails, slugs and bivalves being treated collectively) or its abundance class (in other taxonomic groups). Bryophytes were treated collectively because hepatics alone did not reach acceptable stress values. Potentially important environmental factors extracted in the previous steps (Table S5) were included to explore their correlations with the ordination axes formed.

To detect drainage-sensitive species and post-drainage colonisers, we first carried out indicator species analyses (ISA [70]) by site type (two groups). To check for additional constraints set by timber-harvesting sensitivity, we performed supplementary ISAs where each site type was split by forest cover (forests vs. cutovers, i.e. four groups) and, finally, forest naturalness (old growth vs. mature; four groups). In assemblage-composition analyses (including ISA), species recorded in 1–2 plots were omitted. The analyses of vascular understory plants only included herbs and dwarf shrubs. In the case of snails, only adults were considered because many juveniles remained unidentified at the species level.

## Results

### Species Richness and Stand Structure

A total of 884 species, including 157 SPEC (species of national conservation concern), were recorded: 333 species of vascular plants, 208 bryophytes (152 mosses and 56 hepatics), and 277 lichens and allied fungi in the 44 plots, and 68 snail species (with 11,041 specimens) in the 40 plots studied (Tables S1, S2, S3, S4). The numbers were slightly higher for drained sites: 775 species (including 127 SPEC), compared to 734 (105) species in natural swamp sites. However, that difference was consistent for vascular plants only (Table 1). Two bryophytes previously considered extinct in Estonia [16] were rediscovered: *Amblystegium humile* (in six plots, mostly cutovers) and *Hypnum fertile* (in an old-growth swamp forest). The snail *Vertigo lilljeborgi* (on a swamp cutover) and the lichen *Thelocarpon intermediellum* (in drained forest and cutover sites [71]) were found for the first time in the country.

**Table 1:** Total number of all species and species of national conservation concern (SPEC) by site types and management types.

Species group	Total no. of species (no. of SPEC)									
	Swamp					Drained				
	Old growth	Mature	Retention	Clearcut	Swamp total	Old growth	Mature	Retention	Clearcut	Drained total
	*n* = 5	*n* = 5	*n* = 5	*n* = 5	*n = *20	*n* = 6	*n* = 6	*n* = 6	*n* = 6	*n = *24
Vascular plants	153(11)	165(10)	211(10)	186(8)	260(19)	185(14)	187(15)	243^2^(14^2^)	213^3^(11^2^)	299^4^(28^3^)
* Herbs*	130(11)	143(10)	188(10)	161(6)	233(17)	163(14)	163(15)	221^2^(14^2^)	187^3^(11^2^)	272^4^(28^3^)
* Woody plants*	23	22	23	25(2)	27(2)	22	24	22	26	27
Hepatics	38(7)	29(2)	18(0)	27(4)	50(10)	36(8)	28(3)	18(1)	20(3)	45(10)
Mosses	97(7)	84(7)	96(9)	81(7)	130(14)	97(11)	82^3^(7^1^)	100^1^(10)	84^2^(7)	124^6^(15^1^)
Lichens	167(42)	137(22)	126(6)	143(16)	237(50)	161^3^(41^3^)	156^1^(29^1^)	143^5^(12^2^)	122^2^(9^1^)	231^9^(51^6^)
* Macrolichens*	44(6)	43(2)	40(1)	53(4)	70(9)	44(5)	43(1)	51^2^(0^1^)	45^1^(0)	67^2^(6^1^)
* Microlichens*	123(36)	94(20)	86(5)	90(12)	167(41)	117^3^(36^3^)	113^1^(28^1^)	92^3^(12^1^)	77^1^(9^1^)	164^7^(45^5^)
Snails	33(6)	43(7)	42(9)	43(5)	57(12)	41(7)	37(6)	35(7)	32(5)	57(13)
Total	488(73)	458(48)	493(34)	480(40)	734(105)	520^3^(81^3^)	490^4^(60^2^)	539^8^(44^4^)	471^7^(35^3^)	756^19^(117^10^)

*n–*number of 2-ha plots studied.

*Note.* To enable direct comparison of swamp and drained sites, the main numbers for drained sites also refer to *n* = 5 for each management type (20 plots in total); the numbers of additional plant, bryophyte and lichen species found from the 6^th^ (north-easternmost, cf. Fig. 1) cluster studied are given in superscript.

**Figure 2:**
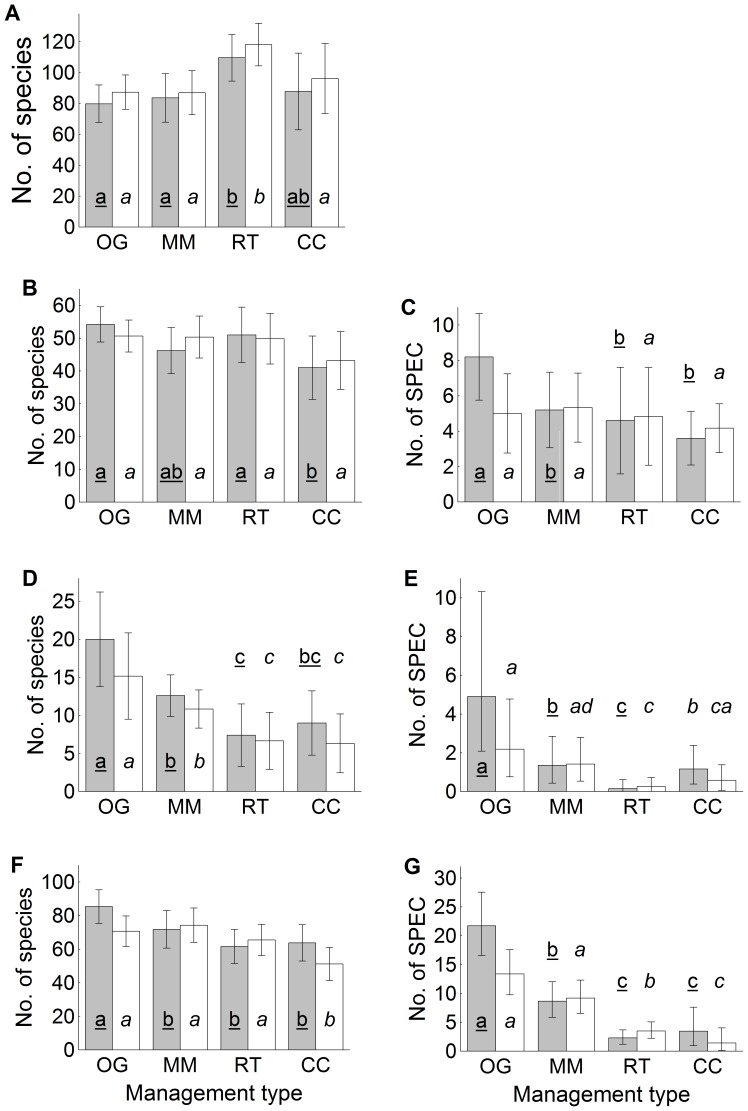
Mean plot-scale (2 ha) species richness and the number of species of conservation concern (SPEC). Only those taxonomic groups that significantly responded to management type are included: vascular plants (A); mosses (B–C); hepatics (D–E); and lichens (F–G). Filled bars are swamp sites and empty bars are drained sites; whiskers are 95% confidence intervals. Significant differences (p<0.05) among management types (OG, old growth; MM, mature managed forest; RT, retention cut; CC, clearcut) according to LSD post-hoc tests are indicated with different letters separately for swamps (underlined) and drained plots (in italics). Differences between drained sites and swamp sites for a given management type were never significant.

On the plot scale, we found clear stand-structural differences between swamps and drained sites, while species richness differed only weakly and depending on timber-harvesting intensity (no main effects of site type across management types; Table 2). Of the four major stand-structural differences (Table S5), drained old-growth forests had significantly lower canopy-tree diversity and CWD continuity, while a combination of relatively low densities of black alder and high densities of Norway spruce was most pronounced in managed forests. Clearcutting always reduced stand-scale species richness of lichens and bryophytes (and retention cuts never differed from true clearcuts; Fig. 2), but vascular plant richness responded (increased) significantly in drained sites only (Fig. 2a). Old-growth swamp forests were distinctly rich in cryptogam SPEC, notably lichens (Fig. 2g). Snails were distributed rather evenly among site-type×management-type groups: on average 277 individuals of 20 species, including 3.2 SPEC, per 3 litres of litter. All taxonomic groups combined, drained plots hosted, on average, 18 SPEC per plot (range 5–36), compared to 25 SPEC in swamps (13–45).

**Table 2:** Split-plot ANOVA on drainage (between-subjects factor) and timber-harvest (within-subjects factor) effects on the 2-ha scale species richness and, separately, on the number of species of conservation concern in the five taxonomic groups studied.

Taxonomic group	Effect						No. of species
	Drainage		Harvest		Harvest×Drainage		
Total number of species	F_1,9_	p	F_3,27_	p	F_3,27_	p	Mean (min.–max.)
Vascular plants	1.6	0.236	7.2	0.010	0.1	0.983	94 (54–135)
Mosses	<0.1	0.879	4.0	0.018	0.6	0.635	48.3 (30–67)
Hepatics	1.9	0.196	17.5	<0.001	0.6	0.642	10.9 (2–27)
Lichens	1.8	0.209	10.2	<0.001	2.9	0.054	67.8 (32–94)
Snails	0.1	0.748	0.6	0.594	0.6	0.597	20.3 (7–29)
**Number of species of conservation concern**							
Vascular plants	0.9	0.677	0.2	0.898	0.1	0.975	3.3 (0–7)
Mosses	0.4	0.546	3.4	0.032	2.0	0.133	5.1 (1–11)
Hepatics	1.2	0.300	120.4	<0.001	1.2	0.338	1.8 (0–10)
Lichens	1.7	0.220	51.4	<0.001	3.5	0.030	8.2 (0–25)
Snails	1.3	0.292	0.6	0.615	0.9	0.435	3.2 (0–6)

*Notes.* Significant effects are presented in detail on Fig. 2. In the tests on snails, the degrees of freedom are 1 and 8 for the drainage effect, and 3 and 24 for the other effects.

Eight stand-structural characteristics correlated significantly with stand-scale species richness in forests, with cryptogam SPEC being clearly most structure-dependent (Table S6). Combining these effects with the site-type (incidence of drainage) effects in general linear models revealed the appearance of a marginal main effect of drainage for lichen SPEC (p<0.1) when either tree species diversity or the volume of logs was accounted for; a similar tendency was observed for hepatic SPEC (p = 0.1) when accounting for the volume of logs.

### Assemblage Composition and Drainage-sensitive Species

**Figure 3:**
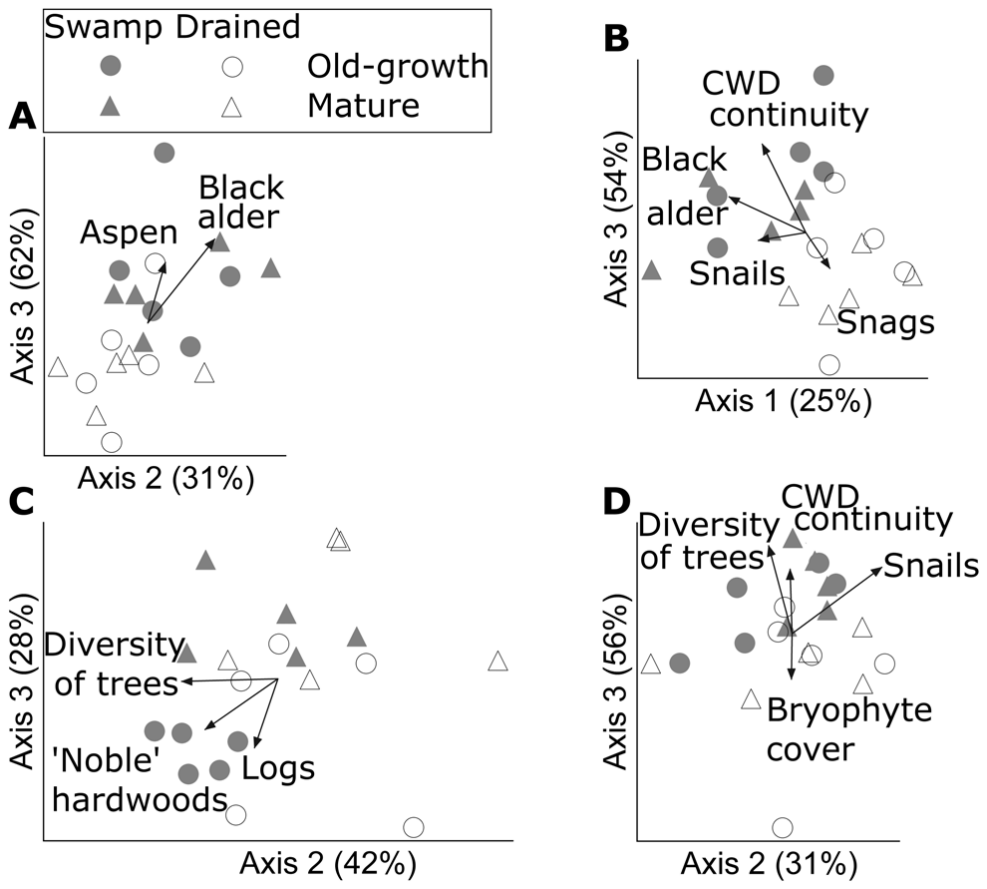
NMS ordination diagrams of the species’ assemblages in forest plots. (A) herbs and dwarf shrubs, (B) bryophytes, (C) lichens, (D) snails. The two most representative axes (% variance explained indicated in titles) of the 3-dimensional solutions and environmental factors correlated with these axes at combined r^2^>0.2 are shown (all factors are listed in Table S5). Note that the factor ‘Snails’ refers to total snail abundance.

Drainage changed assemblage composition most clearly in forests (Table 3) and there was tentative evidence for elevated logging sensitivity in the ground vegetation (both in vascular plants and bryophytes, the mature forest vs. clearcut contrast was only significant in drained sites; MRPP-tests: p<0.005). The assemblages of old-growth vs. mature forests only differed in the case of hepatics and lichens in swamp forests (MRPP-tests: p = 0.028 and p = 0.005, respectively), while no significant differences between assemblages in retention cuts and clearcuts were observed. Drainage-sensitive structural characteristics (CWD continuity and at least one variable describing tree-species composition) were significantly related to assemblage composition in each taxonomic group (Fig. 3, Table S5).

**Table 3:** The significance of assemblage differences between swamp and drained plots by taxonomic group and management type (MRPP tests).

	Swamp-drained contrast, p-value			
	Old growth	Mature	Retention cut	Clearcut
Herbs and dwarfshrubs	0.013	0.003	0.324	0.038
Lichens	0.016	0.053	0.045	0.057
Mosses	0.012	0.001	0.152	0.374
Hepatics	0.004	0.021	0.032	0.763
Snails	0.263	0.012	0.281	0.221

We detected a total of 130 indicator species for swamp sites (Table 4). Among those apparently drainage-sensitive species, forest-dwelling lichens formed the largest group with 42 species. Lichens and bryophytes were also most sensitive to harvesting of swamp forests: all the 16 lichen SPEC and six of eight bryophyte SPEC, which had significant indicator values for swamps, were typical of forests and none were typical of cutovers (Table 4). Seven SPEC were strictly concentrated to old-growth swamp forests and thus suffer from both studied types of management: the hepatic *Geocalyx graveolens*, macrolichens *Lobaria pulmonaria* and *Menegazzia terebrata*, and microlichens *Arthonia byssacea*, *A. leucopellaea*, *A. vinosa* and *Pertusaria flavida*. Drained plots hosted three characteristic bryophyte and four lichen SPEC, but none of those were confined to old growth.

**Table 4:** Numbers of “indicator species” (indicator species analyses: p<0.05, uncorrected for multiple tests) by taxonomic group and habitat type (SW–swamp; DR–drained).

Habitat type	Set^a^	No. of indicator species (incl. no. of species of conservation concern)					
		Herbs and dwarf shrubs	Mosses	Hepatics	Lichens	Snails	Total
SW all types	A	14(1)	5(1)	1(1)	4	1	25(3)
SW forest	B	10	7(2)	6(3)	31(10)	2	56(15)
SW old-growth	C	0	2	3(1)	8(6)	0	13(7)
SW mature	C	1	1	0	3	0	5(0)
SW cutover	B	14(1)	3	0	10	4(1)	31(2)
DR all types	A	3	6(1)	0	1	0	10(1)
DR forest	B	11	4	3(2)	8(3)	0	26(5)
DR old-growth	C	1	3	0	0	0	4(0)
DR mature	C	6	5	1	6(1)	0	18(1)
DR cutover	B	34	5	0	13	2	54(0)
Total		94(2)	41(4)	14(7)	84(20)	9(1)	242(34)
% of species		28	27	25	31	13	27

*Note.* ^a^three sets of hierarchically arranged analyses were performed, using different resolution for habitat grouping: A, two site types (management types not distinguished); B, 2 site types×forests vs. cutovers (4 groups); C, 2 site types×old growth vs. mature forest (4 groups; only forest sites included). For each species, only the highest habitat resolution is reported (results from C were additionally compared to B to remove species that occurred both in forests and cutovers), i.e., the numbers are exclusive. For example, in addition to three hepatics typical of old-growth swamp forest there were six indicator species for swamp forest in general and one species for swamp sites in general.

Herbs showed an opposite pattern–there were more indicator species for drained sites, particularly for cutovers (Table 4), and most of these are widespread species. Both characteristic species of old-growth drained forests–the “indicator” herb *Epilobium montanum* and the shrub *Daphne mezereum*–are common in the country, while the analysis detected no herb species typical of old-growth swamp forests. Only two herb SPEC had a significant indicator value–*Dryopteris cristata* for swamp cutovers and *Carex disperma* for swamps in general. Sedges (*Carex* spp.) were a species group that collectively tended to suffer from drainage: there were seven indicator species for swamps and only two for drained sites (Table S1).

Seven of the nine significant indicators among snails preferred swamps, but only two of them were confined to forests (Table 4). The only indicator SPEC, *Carychium minimum*, was most abundant in swamp cutovers (55% of individuals collected; Table S4).

### The Assemblages and Conservation-value Species in Drained Sites

A total of 158 species were only observed in drained sites and ISA distinguished 113 indicator species (Table 4); the latter can be broadly considered post-drainage colonisers (including large expansions within stands). The largest indicator group (34 species) was vascular understory plants colonising drained cutovers, which had in total (considering also rarities that did not pass the ISA) 33 vascular plant species not found elsewhere. Most of these plants (Table S1) are characteristic of dry meadows or disturbed areas in Estonia. Drained cutovers also had 14 species of indicator lichens (mostly on deciduous tree regeneration, logging residues and stumps). Among mosses, *Brachythecium* spp. appeared as a drained-site preferring group, with species distributed across different management types (Table S2). However, only seven “colonisers” were SPEC: four bark or wood-dwelling lichens (*Chaenotheca stemonea*, *Micarea hedlundii*, *M. tomentosa*, *Pertusaria pupillaris*) and two hepatics (*Anastrophyllum hellerianum*, *Nowellia curviflora*) confined to forests, and the ground and deadwood-dwelling moss *Campylium halleri* in both forests and cutover sites (Tables S2–S3).

Drained forests had distinct assemblages in mature managed, rather than in old-growth stage (Table 4). Most of the 18 indicators of drained mature stands were (i) sparsely growing plants of eutrophic forests–some ground-layer bryophytes (e.g., mosses *Brachythecium oedipodium*, *B. reflexum*, *Plagiothecium curvifolium*, *Rhodobryum roseum*), herbs (such as *Dryopteris filix-mas*, *Fragaria vesca*, *Mycelis muralis*) and the grass *Milium effusum*; or (ii) lower-trunk inhabiting lichens (e.g., *Lepraria jackii* and *Cladonia chlorophaea*; also the only SPEC in such forests, *Pertusaria pupillaris*). These same two types of indicator species were represented among the general drained-forest indicators (old-growth and mature stands pooled), for instance, herbs *Actaea spicata*, *Anemone nemorosa* and *Oxalis acetosella*, the increasingly dominant moss *Hylocomium splendens*, and various crustose lichens.

We distinguished 20 forest-dwelling SPEC relatively indifferent to drainage (present in at least three drained forest plots and neither indicators of drained nor swamp forests): four vascular plants (*Dactylorhiza fuchsii*, *Huperzia selago*, *Poa remota*, *Stellaria longifolia*), four mosses (*Homalia trichomanoides*, *Plagiomnium undulatum*, *Plagiothecium latebricola*, *Ulota crispa*), the hepatic *Scapania apiculata*, six microlichens (*Chaenotheca chlorella*, *C. trichialis*, *Lecanactis abietina*, *Reichlingia leopoldii*, *Thelotrema lepadinum*) or allied fungi (*Chaenothecopsis haematopus*), and five snails (*Acanthinula aculeata*, *Aegopinella pura*, *Macrogastra ventricosa*, *Perforatella bidentata*, *Vertigo ronnebyensis*). The snails in particular (as well as several other species) were, however, often present in cutover sites as well (Tables S1, S2, S3, S4). Additionally, there were 46 rare SPEC (recorded in 1–3 plots) with at least one record from drained forests; 20 such species (incl. 13 lichens) were only recorded in old growth.

## Discussion

The general patterns emerging from our study were: (1) drainage (ditching) impacts on non-aquatic biodiversity interact with timber harvesting; these impacts also depend on taxonomic group and are manifested in species composition rather than in species richness. Although the effects on stand-scale species richness may be larger than observed (due to underestimations increasing with species richness; see Data collection), they were relatively much smaller than timber harvesting impacts; (2) major drainage influence, notably on cryptogam assemblage composition, is caused by changes in stand microhabitat structure; (3) drained forests are novel ecosystems that can host many threatened species, which are not, however, specialist species of natural site types. As discussed below, these patterns highlight a need for three types of science-based management tools to mitigate the drainage effects in forest landscapes: (i) silvicultural techniques to maintain critical structural complexity in drained stands; (ii) context-dependent spatial analysis and planning of drained landscapes to balance stand-scale losses and gains of biodiversity; and (iii) lists of focal species to monitor and guide drainage practices.

### Draining and Timber-harvesting Impacts on Biodiversity Combine

In practical forest management, drainage is usually accompanied with logging (see Introduction). We found that such combined effects are inherently complex and dependent on taxonomic group, which implies that there is no simple way to assess “the drainage impact on biodiversity”. However, the complexity can be organised based on empirical research. In our study system, we can broadly distinguish four combinations of drainage influence discussed below: in forests vs. in cutovers, and separately for vascular plants and snails vs. for cryptogams. In the whole dataset, we detected only four SPEC that responded to drainage but not to clearcutting (three wetland species, and one post-drainage “colonist”, the moss *Campylium halleri*). All those species inhabited the ground layer, and the wetland species apparently mitigated post-harvest microclimatic change by using protected microhabitats. Thus, a hepatic of conservation concern, *Conocephalum conicum*, along with some common plants (e.g., the herb *Myosotis scorpioides* and the hepatic *Marchantia polymorpha*), ubiquitously inhabited muddy depressions–treefall pits in forests and tractor ruts in cutovers. The threatened small sedge *Carex disperma* and some moisture-dependent mosses (e.g., *Calliergon giganteum*, *Drepanocladus aduncus* and *Aulacomnium palustre*) survived in small wet patches, which were maintained by harvest disturbance and protected from desiccation by proliferating tall vegetation surrounding the patch.

1. Cryptogams (bryophytes and lichens) in closed stands were most clearly affected by drainage, notably as revealed by indicator species analyses. Old swamp forests are known to be cryptogam diversity hotspots [72], [73] but we highlight that, even if such stands are not logged, they can slowly lose species of conservation concern (notably lichens and hepatics) after ditching. The sensitivity of swamp-forest cryptogams was further confirmed by the fact that the clearcutting origin of forests (i.e., the difference between old growth and mature stands) mattered more in the swamp sites than in the drained sites–which is in concurrence with what Rosenvald et al. [40] reported for birds. These findings should be robust, although we did not sample tree canopies (but many species were recorded on fallen trees or as litterfall), because moisture-dependent species typically grow on or near the forest floor (e.g. [74]). We thus conclude that (i) where old swamp forests still exist, they constitute priority targets for reserve establishment (see also [75]), while (ii) in the forests that are already drained (notably in mid-aged stands in reserves) a major management issue is whether vulnerable key structures (woody vegetation and deadwood) and their heterogeneity could be maintained or restored [76].
2. In cutovers, lichen species composition (but not species richness or the number of SPEC) responded to drainage as well, and this effect was also observed for hepatics in retention cuts (Table 3). However, only one cryptogam SPEC tended to prefer cutovers (the rediscovered moss *Amblystegium humile*, both in swamp and drained sites), which indicates that most cutover “colonists” (notably lichens on deciduous tree regeneration, logging residues and stumps) are widespread and common species. Because cryptogam richness declined on both swamp and drained cutovers (Fig. 2), their main management questions at final felling are not related to drainage but to protecting important habitat structures. Studies in comparable site types indicate that those structures include, in particular, live trees of different species [77], large-sized downed deadwood [78], standing dead trees and windthrow mounds [46]. In addition to substrate provision, such structures may protect ground-layer cryptogams from mechanical disturbance [79]. Protection from desiccation probably matters less in the eutrophic site types studied where clearcutting induces secondary paludification and the proliferation of tall vegetation (graminoids in swamps; *Rubus idaeus* and *Epilobium angustifolium* in drained sites).
3. Cutover sites formed distinct habitats for vascular understory plants and snails, with several SPEC recorded. In vegetation, such a pattern is known from various forest types, and a likely mechanism is the non-linear effect of canopy opening on many species that only appear at >80% tree removal [80], [81]. Vegetation reorganisation affects those snail species that feed on live plants or plant litter, but specialised herbivorous snails can also modify plant communities, especially seedlings [67], [82]. Drainage influences on these assemblages were more subtle and apparently modified by the extent of secondary paludification after logging, given that snails and plants alike respond to soil mineral content and moisture [83]. This secondary paludification is caused by decreased evapotranspiration (particularly interception) by trees [84], [85] and it promotes open-mire vegetation, notably the invasion of sphagna [86]. Tree retention probably mitigated paludification more in drained sites, as revealed by an increase in plant species richness (Fig. 2a) and drainage-dependent vegetation composition in retention cuts, in contrast to the homogenised assemblages in swamp vs. drained sites after complete clearcutting (Table 3). However, the two indicator SPEC preferring swamp cutovers (fern *Dryopteris cristata*, snail *Carychium minimum*) and (taken collectively) rare wetland SPEC absent from forests (e.g. plants *Carex irrigua, C. rhynchophysa*, *Iris sibirica* and *Stellaria uliginosa*, the snail *Vertigo angustior*) occurred at similar frequencies both in clearcuts and in retention cuts. Therefore, in this early stage of succession, various natural and drained cutovers can provide some habitat for rare open-wetland plants and snails, and their habitat quality is not reduced by scattered trees vital for cryptogams.
4. Among vascular understory plants and snails, we detected no forest-preferring SPEC and no indicator species of old-growth swamp forests, but drainage modified their assemblage composition (except in snails in old growth; Table 3). In snails, the loss of small bodies of water and seasonal floods [20] may be responsible for the reduction of some species (water snails, *Pisidium* clams, *Succineidae*, *Cochlicopa nitens*, *Zonitoides nitidus*; see [87]) and the pronounced drainage influence in mature stands corresponds to changes in tree-layer (see below). The latter effect was probably both litter and understory-mediated, given that detritivorous species dominated in the samples. The absence of drainage effect on snail assemblages in old growth may be a result of factor interactions, such as an increase in important food plants (e.g. *Urtica dioica*; [88]) versus a decrease in tree-species diversity.

### Microhabitat Structure Mediates Drainage Impacts in Closed Stands

Our main evidence that stand structure mediates the major long-term effects of forest drainage (notably on the most drainage-sensitive assemblage–cryptogams in closed stands) included habitat requirements shared by drainage-affected species (indicator species analysis) and the consistency of drainage-affected stand-structures in explaining assemblage compositions (ordination). Hence, selective removal and creation of microhabitats is a major mechanism, which assembles species in novel ecosystems and can be used to manage their biodiversity (e.g., [89], [90]). Because the most influential features–canopy-tree composition and CWD continuity–develop very slowly [65], [91], there is a long time delay until structure-dependent species respond. We warn that such delayed influence on what might appear as “resistant” biodiversity in the first generation of drained forests can be most detrimental in the long run.

In the tree layer, the economically desirable increase of Norway spruce was accompanied with decreases in the black alder and “noble” hardwoods. The latter was statistically non-significant but the tendency was clear (Table S5; note our small samples) and has been reported before [61]. Notably, European ash (*Fraxinus excelsior*) is a successful late-seral species regenerating in canopy gaps of various wet and moist forests in the Baltic region [45], [92] but drainage appeared to inhibit this process. In terms of tree diversity, the impacts magnified along succession: natural swamps became more diverse by the old-growth stage, while the increasing spruce dominance impoverished aging drained forests (Table S5). This may reflect positive feedback in the regeneration of the shade-tolerant Norway spruce on decayed peat.

Lichens were most affected by the tree-layer transformation, although at least one canopy variable (notably tree-species diversity) co-varied with every assemblage (Fig. 3, Table S6). Six of the seven SPEC typical of old-growth swamp forests were epiphytic lichens that typically inhabit large old trees in long-term forests ([93], [94], [95]; but note that they can survive low-level selection cutting that retains most tree cover and the host trees in some regions [51]). “Noble” hardwoods provided particularly important habitats for swamp-forest lichens, with many rare epiphytes that prefer such trees only found there (e.g. *Agonimia allobata*, *Arthonia byssacea*, *Cheiromycina* spp., *Fellhaneropsis vezdae*, *Mycobilimbia hypnorum*, *Opegrapha viridis*, *Pertusaria flavida*, *Pyrenula laevigata*). Historically, many such species probably had strongholds in floodplain forests, which have been devastated in Europe [96], while “noble hardwoods” in general have suffered a manifold reduction in Estonia due to timber harvesting [45]. Hence, drainage can degrade the remaining refuge habitats of such lichens in swamps.

Unexpectedly, spruce abundance was not related to assemblage characteristics in any taxonomic group studied. This extends previous observations in the Baltic forests that the spruce stands established in areas formerly under deciduous wetlands neither keep the original ground vegetation nor establish that of spruce swamps [61], and that artificial planting of spruce does not introduce new fungal species to naturally deciduous-dominated landscapes [97]. Spruce was probably present (at least in understory) in sufficient numbers to host specific species in most stands and/or its substrate value differed in swamps and drained forests. For example, its higher trunk-scale richness of epiphytic lichens in swamps [98] could compensate, on the stand scale, for abundant, but smooth-barked and heavily shaded, trees in drained forests. The influence may also depend on other tree species; for example, snails respond to the variation from broad-leaved to needle-dominated litter [99]. This might explain the distinct drainage impact on snails in drained mature stands, which experienced the greatest increase of spruce and a simultaneous loss of black alder. Changed litter composition may perhaps even limit some snail populations; for example, *Ruthenica filograna* prefers abundant alder leaves in wintering sites [100] and we only found it in swamp sites.

CWD continuity (measured as decay-stage diversity) was significantly related to the composition of every assemblage studied. For deadwood-dwellers (these have highly variable requirements [101]), this primarily reflects microhabitat diversity–indeed, it contributed to species richness in bryophytes and lichens only (Table S6). For understory plants and snails, “CWD continuity” most likely revealed general habitat heterogeneity, such as small-scale interspersion of logs, bare ground, and the microtopography created by old treefalls. Continuity *s. str.* (duration of a relatively stable disturbance regime [65]) was probably unimportant because old growth (particularly in the native swamp type) did not appear to have special value for any herb or snail species. Among bryophytes, sphagna are perhaps the most continuity-dependent [102], but we only found one old-growth species in this group (*Sphagnum capillifolium*; Table S2). Rather, bryophytes also benefited from ground-level heterogeneity [103] and/or the variability in shade conditions important for lichen diversity (e.g. [46]).

In management terms, the slow stand-scale reorganisation of forest structure after ditching differs from the abrupt and profound tree-scale effects of timber harvesting. When ditches are filled for restoration, a similarly slow recovery follows; thus some restoration cutting may be necessary to speed up the processes in reserves [76]. However, in most forests drained for timber harvesting, ditches continue to serve the economic aims and distinct drainage-mitigation measures may be required for structure-dependent biodiversity. Such measures have yet to be elaborated and tested, but our study highlights tree-species diversity and dead wood as crucial issues. A central question is how to sustain regeneration of drainage-sensitive tree species, for example, by retaining mature seed trees, creating gaps among the regeneration of the main tree species, site preparation, or even artificial regeneration. Once such trees are present, they should be carefully retained at subsequent harvesting operations. The focal tree species vary regionally: while black alder and “noble” hardwoods were of concern in our study (hemiboreal Europe), slowly-grown Norway spruce, European aspen (*Populus tremula*) and goat willow (*Salix caprea*) are key species only a few hundred km further north [98], and thinleaf alder (*Alnus incana subsp. tenuifolia*) has been highlighted for the hemiboreal riparian forests of North America [104].

Tree-species diversity is also important for dead-wood management, which could additionally aim at higher-than-average amounts. We observed that abundant logs tended to mitigate–probably by substrate provision and enhanced ground-habitat heterogeneity–drainage-related reduction in the stand-scale richness of lichen and hepatic SPEC (see [73] for a similar compensatory effect on stand continuity). In fact, five of the seven cryptogam SPEC preferring drained forests (but also occurring in swamps) were confined to well-decayed fallen trunks: the hepatics *Nowellia curvifolia* and *Anastrophyllum hellerianum*, the xerophilous moss *Ptilium crista-castrensis*, and epixylic lichens *Micarea tomentosa* and *M. hedlundii*. Because mid-aged and mature drained forests often have increased susceptibility to windthrow, the initial management step might be simply to reduce windthrow removal.

### Drained Forests for Biodiversity

The compositional differences between swamp and drained sites were large, but rather balanced: ISA distinguished 130 species of a total of 884 species as disappearing and 113 species as appearing post drainage, and 158 species were only observed in drained sites. These numbers certainly include errors, such as overestimating “turnover” with the chronosequence approach (perhaps even some differences of historically drained swamps from those survived) and underestimating it due to the low statistical power of ISA in the case of rare species that are of major conservation interest. Nevertheless, the magnitude of these numbers indicates how laborious systematic conservation assessment of novel forest ecosystems is even for a few stands, while the ultimate aim might be to follow the affected populations on the landscape-scale. In Estonia, the relatively high level of connectedness and diversity of forests [36] probably contributed to the colonisation of drained sites, which can be slower in more impoverished regions (cf. [105]). Broad-scale considerations are fundamental for understanding the functioning of novel ecosystems [106], but they are lacking in the whole emerging field of systematic conservation assessment of such ecosystems (e.g., [107], [108]) and remain only speculative in our study as well.

What we can conclude, based on the intensive documentation of species diversity across multiple taxonomic groups, is that the “colonists” of drained sites represent typical post-disturbance, successional, and generalist species that readily occupy cutovers and managed forests. Thus, the only snail characteristic of drained plots, *Macrogastra plicatula*, preferred cutovers (and certainly does not indicate woodland key habitats as suggested by Pilāte [109]), and the invasive *Arianta arbustorum* was also most abundant in drained sites. The ground vegetation in closed stands had not only started to resemble that of meso-eutrophic and eutrophic mineral-soil forests (see [26], [110]), but it also hosted some putatively forest management-sensitive plants of those site types (such as *Actaea spicata*, *Dryopteris filix-mas* and *Huperzia selago* [37]). However, drained old-growth stands hosted very few specific species and their species richness seldom exceeded that of mature managed stands. This indicates that such stands do not provide quality habitats for true old-growth species of drier forest types and/or such species cannot colonise drained forests within two forest generations. Whether that situation might still improve later is important particularly for planning and managing the reserves that contain drained forests, but finding appropriate study sites (with a long drainage history and no timber harvesting) is a challenge.

Although drained forests failed to provide quality habitats for many swamp species and for old-forest species of drier site types, they appeared valuable for less site type-specific species. Those included many rarities and old-forest specialists (of the 127 SPEC recorded, 84 were present on just 12 ha of drained old growth) with all the taxonomic groups being well represented (Table 1). In addition to lichens and bryophytes that depend on specific stand structures and their heterogeneity (see above), herbs formed a large group of conservation interest. Many such plant SPEC inhabited drained cutovers and may be negatively affected by soil scarification and artificial regeneration with monocultures. While scarification (which was not practiced in our study sites) may be useful on mineral soil for restoring some plant populations with long-term persistent seed banks [81], it is unclear whether such seed banks exist in the decomposing peat soils. In a limited extent this practice could benefit some rare ruderals, but attention should be paid to maintaining downed dead wood, which provides vital habitats for cryptogams and is highly vulnerable to scarification [111]. Another vascular-plant group of conservation value were shade-tolerant herbs in closed stands, notably several orchids (see [33]). Finally, it is likely that drainage ditches (not sampled by us, but see [112]) provide novel habitats for some wetland species and shade-tolerant plants, such as *Carex remota*.

Given the high biodiversity of wet forests and the profound impacts of drainage on species composition (not necessarily on species richness), there is a need for selected species for guiding adaptive management of artificial drainage towards more nature-friendly directions. Those focal species should be well detectable and represent different ecological groups, threat factors and, as a consequence, different spatial scales [113]. The immediate conservation concern is the loss of specific biodiversity of natural wet forests, especially those confined to old growth. Based on our analyses, we propose the following cryptogams as a starting point for relevant focal-species lists: two epiphytic lichens (macrolichen *Menegazzia terebrata*, microlichen *Arthonia vinosa*; see also [93] and [94]), the hepatic *Riccardia palmata* on well-decayed fallen trunks, and highly moisture-dependent hepatic *Trichocolea tomentella*. Their management should be performed on the scale of stand mosaics within landscape, with the aim of sustaining a sufficient number of viable stand-scale populations, so that the colonisation potential for new stands is also retained. Along with the testing of the effectiveness of those focal species, we call for collecting and analysing extensive empirical datasets in other regions of the world, notably on taxonomic groups not included in our study. This is important because of the apparently poor performance of traditional “experience-based” lists of focal species for forest management [46]. The same impression was obtained in our study when comparing the results with such lists previously published (e.g. [37]).

## Supporting Information

Table S1 Number of plots occupied by species of vascular understory plants in swamp and drained sites by management type, and significant habitat preferences according to indicator values for herbs and grasses.
(XLS)

Table S2 Number of plots occupied by bryophyte species in swamp and drained sites by management type, and their significant habitat preferences according to indicator values.
(XLS)

Table S3 Number of plots occupied by lichens and allied fungi in swamp and drained sites by management type, and their significant habitat preferences according to indicator values.
(XLS)

Table S4 Number of plots occupied by snail species (no. of individuals in brackets) in swamp and drained sites by management type, and their significant habitat preferences according to indicator values.
(XLS)

Table S5 Means of environmental variables in swamp and drained sites by management type.
(XLS)

Table S6 Significant correlations between biodiversity and environmental factors.
(XLS)
